# Effects of Emotion-Focused Therapy on Weight Restoration and Emotion Regulation in Adolescents With Anorexia Nervosa

**DOI:** 10.62641/aep.v54i4.2290

**Published:** 2026-08-15

**Authors:** Linfeng Liu, Nongmei Cheng, Qing Feng

**Affiliations:** ^1^Department of Child and Adolescent Psychiatry, Chongqing Mental Health Center, 401147 Chongqing, China

**Keywords:** emotion-focused therapy, adolescents, anorexia nervosa, weight, emotion regulation

## Abstract

**Background:**

To investigate the effects of emotion-focused therapy (EFT) on weight restoration and emotion regulation in adolescents with anorexia nervosa (AN).

**Methods:**

This retrospective study included 188 adolescents with AN treated at Chongqing Mental Health Center between January 2023 and December 2025. According to treatment methods, patients were divided into a control group and an EFT group. After screening based on inclusion and exclusion criteria, 60 patients in each group were included in the final analysis. The control group received routine nutritional support, cognitive behavioral therapy, and medication when necessary, while the EFT group additionally received EFT. Both groups were treated for 12 weeks. Body mass index (BMI), nutritional indicators, Eating Attitudes Test-26 (EAT-26), Trait Meta-Mood Scale (TMMS), Generalized Anxiety Disorder-7 (GAD-7), and Children’s Depression Inventory-2 (CDI-2) scores were compared between the two groups. Safety outcomes were retrospectively collected from electronic medical records. Multiple linear regression analysis was performed to evaluate the independent association between EFT and BMI improvement.

**Results:**

After 12 weeks of treatment, BMI and nutritional indicators in the EFT group were higher than those in the control group, while the EAT-26 scores were significantly lower (all *p* < 0.05). At weeks 4, 8, and 12, TMMS scores in the EFT group were significantly higher, while GAD-7 and CDI-2 scores were significantly lower than those in the control group (all *p* < 0.05). No significant between-group differences were observed in refeeding syndrome or readmission rates (all *p* > 0.05). Multiple linear regression analysis showed that EFT remained independently associated with higher BMI at week 12 after adjustment for baseline BMI, age, sex, and olanzapine use (β = 0.382, *p* < 0.05). Moderate-to-large effect sizes were observed for major outcome measures (Cohen’s d = 0.77–1.23).

**Conclusions:**

EFT combined with routine nutritional support and psychotherapy may be associated with improved weight restoration, emotional regulation, nutritional status, and reduced anxiety and depressive symptoms in adolescents with AN. However, due to the retrospective single-center design and limited sample size, further prospective studies are warranted to confirm these findings.

## Introduction

Anorexia nervosa (AN) is an eating disorder characterized by self-starvation, excessive weight loss and malnutrition. Its incidence among adolescents is rising year by year [1]. Patients with AN often exhibit symptoms such as excessive concern about weight, extreme restriction of energy intake and body image disorder [2], which not only leads to severe psychopathological symptoms, but also often causes life-threatening complications such as endocrine disorders, cardiovascular problems and osteoporosis [3]. A study reported that about 50% of adolescents with AN can achieve ideal weight after treatment, 30% get improved, but the remaining 20% have a poor prognosis due to late onset, long disease duration or comorbidities [4]. Therefore, early intervention is particularly important for adolescents with AN. Weight restoration and emotional regulation ability are considered key indicators of treatment efficacy [5].

Traditional treatments mainly include nutritional rehabilitation and cognitive behavioral therapy (CBT). Nutritional rehabilitation aims to comprehensively assess the patient’s physical condition and formulate a reasonable dietary recovery plan through the cooperation of physicians and nutritionists to correct malnutrition [6]; while CBT focuses on helping patients change cognitive biases, popularize nutritional knowledge, and master scientific weight management methods [7]. However, these methods have certain limitations. For example, although nutritional rehabilitation can improve the patient’s physical condition, it is difficult to solve psychological problems; while CBT is effective in the short term, it has limited effect on patients with long-term disease or serious psychological problems. Therefore, exploring more comprehensive treatment methods is still an important direction in current research.

Emotion-focused therapy (EFT) originated from humanistic psychology, and its core theory emphasizes the key role of emotion in the individual psychotherapy process [8]. This therapy advocates improving mental health by enhancing an individual’s cognition, acceptance and expression of emotions, thereby promoting the ability of emotion regulation. In recent years, EFT has been widely used in the treatment of various mental illnesses such as depression, social phobia, and post-traumatic stress disorder [9]. In the field of eating disorder treatment, EFT has received particular attention. For example, studies have shown that EFT can help patients identify and express repressed emotions and reduce excessive dieting behavior caused by emotional dysregulation [10].

This study retrospectively analyzed the clinical efficacy of EFT in adolescents with AN and compared it with conventional therapies.

## Materials and Methods

### Research Subjects

This retrospective study initially extracted clinical data of 188 adolescents with AN from the Department of Child and Adolescent Psychiatry of Chongqing Mental Health Center from January 2023 to December 2025. Patients were divided into an EFT group (n = 95) and a control group (n = 93) according to whether they received additional EFT. The selection of treatment methods considered the patient’s condition, their own and their guardian’s wishes, as well as the doctor’s judgment.

In the EFT group, 12 patients were excluded due to incomplete clinical data, 8 due to non-first onset, 6 due to comorbid psychosis, 5 due to cognitive impairment, and 4 due to severe physical illnesses, resulting in a final inclusion of 60 patients.

In the control group, 11 patients were excluded due to incomplete clinical data, 6 due to non-first onset, 6 due to comorbid psychosis, 5 due to cognitive impairment, and 5 due to severe physical illnesses, resulting in a final inclusion of 60 patients.

Inclusion criteria: age 13–17 years; meeting the diagnostic criteria for AN in the fifth edition of the Diagnostic and Statistical Manual of Mental Disorders (DSM-5) [11]; body mass index (BMI) below the 5th percentile for their age and gender, with reference to the World Health Organization (WHO) growth reference for school-aged children and adolescents [12]; accompanied by physiological changes such as malnutrition, hypotension, tachycardia, and electrolyte disturbance; first onset of AN; disease duration of more than 3 months; and all patients completed 12 weeks of treatment and had complete baseline and follow-up data.

Exclusion criteria: patients with severe psychosis (e.g., schizophrenia or bipolar disorder) or other eating disorders, cognitive impairment, drug or alcohol dependence, or severe physical illness (e.g., primary gastrointestinal, liver or kidney disease, hematonosis).

### Treatment Methods

#### Control Group

The control group received standard treatment including routine nutritional rehabilitation, CBT, and medication when necessary.

(1) Nutritional rehabilitation: A progressive refeeding program was developed based on routine clinical practice in our center and was generally consistent with published recommendations [6]. In the early stage of refeeding, the primary goals were correction of dehydration and electrolyte imbalances. Initial daily energy intake was set at 1400–1500 kcal and increased by 300–400 kcal every 3–4 days. During the first 3 days of refeeding, electrolyte levels of potassium, phosphorus, calcium, and magnesium were monitored daily, and supplemented as required. In the recovery stage, energy intake was increased to 2000–2500 kcal/day, with a target weight gain of 0.5–1 kg per week. In the maintenance stage, patients were discharged and continued treatment at home, where individualized dietary plans were prescribed, with a daily energy intake of 70–100 kcal/kg.

(2) CBT: ①Cognitive set shifting: Patients were guided to recognize that physical appearance and body weight are not direct indicators of self-worth. With parental assistance, photographs taken when the patient was at a healthy weight were reviewed, and positive feedback was provided to enhance self-confidence. Patients were also educated about the medical risks of AN, including malnutrition, impaired immune function, and potentially life-threatening complications. ②Identify and address the causes of the patient’s low self-esteem. Therapists explored potential contributors to patients’ low self-esteem, such as experiences of teasing or bullying related to appearance or body weight. When school-related psychosocial stressors were identified, clinicians collaborated with parents and school staff to help address and mitigate these factors. ③Parental guidance and family involvement: A comprehensive psychological assessment of the family was conducted, followed by family-based interventions under the guidance of a psychologist. Sessions were held twice weekly, each lasting one hour. Parents were trained to improve communication with their children, provide emotional support and encouragement, attend to daily needs, and better understand patients’ emotional experiences. Parents were advised to avoid blame or coercive feeding practices, to monitor eating-related and compensatory behaviors, to discourage behaviors such as self-induced vomiting or purging, and to reinforce positive behavioral and emotional changes [13].

(3) Medication: Patients who did not respond to nutritional support after one week were given olanzapine. The dose started at 2.5 mg/d and increased as needed by 1.25–2.5 mg per day. The maintenance dose ranged from 2.5 to 10 mg/d and was gradually reduced as the patient approached their treatment goal weight [14].

#### EFT Group

The EFT group received additional EFT in addition to the standard treatment, twice a week for one hour each time. All therapists participating in this study possessed nationally recognized psychotherapist qualifications and had received EFT- related training.

The EFT treatment process can be divided into three stages [15]. Individualized adjustments were allowed according to patients’ emotional responses and treatment progress, however, the overall treatment framework and session frequency remained consistent across patients.

(1) De-escalation stage (Weeks 1–4): Adolescents with AN often have alexithymia and are unable to correctly identify and express emotions. During this stage, therapists established a secure and trusting therapeutic alliance through empathic attunement. Patients were guided to identify and differentiate their current negative emotional states, and therapists assisted in labeling and clarifying specific emotions. For example, statements such as “I am too ugly to deserve friends” were conceptualized as self-criticism, whereas fears of being ridiculed if weight was not controlled were identified as catastrophic thinking. Therapists further helped patients explore the underlying sources of these emotions, identify associated unmet needs or adaptive emotions, and develop constructive coping strategies to interrupt maladaptive emotional cycles.

(2) Restructuring stage (Weeks 5–8): The empty-chair dialogue technique was employed to facilitate emotional processing and cognitive-emotional restructuring. Patients were asked to imagine a significant conflicting figure (e.g., a peer who had bullied them) seated in an empty chair and to verbally express their emotions toward the imagined person. If emotional arousal became excessive, the therapist paused the intervention until the patient regained emotional stability. Patients were then guided to experiment with alternative responses, such as assertive self-expression or self-protective coping strategies, in order to experience adaptive emotional responses and gradually reduce self-directed hostility.

(3) Adaptation and consolidation stage (Weeks 9–12): In the final stage, patients were encouraged to integrate newly developed adaptive emotional experiences into daily life. Therapists supported patients in consolidating positive emotional states, strengthening self-identity and self-confidence, reconstructing a healthier body image, and reducing maladaptive behaviors such as excessive dietary restriction and self-induced vomiting.

Both groups were treated for 12 weeks.

### Observation Indicators

#### Baseline Data

Baseline data were extracted from the hospital’s electronic medical record system, including gender, age, duration of illness, BMI, family structure, medication use, anxiety level (Generalized Anxiety Disorder-7 (GAD-7) score ≥15 indicates severe anxiety) and depression level (Children’s Depression Inventory-2 (CDI-2) score ≥20 indicates severe depression). Baseline demographic and clinical variables were compared between groups to evaluate group comparability.

Outcome data at baseline and at weeks 4, 8, and 12 after treatment were extracted from electronic medical records. Questionnaire scores had been routinely recorded during clinical care using self-report scales completed by the adolescents. When necessary, medical staff provided clarification to ensure that patients understood the questionnaire items.

#### Body Weight and Nutritional Indicators

Data on body weight, height, and nutritional biomarkers at baseline and week 12 were extracted from electronic medical records. BMI was used as the primary indicator of weight restoration and was calculated as weight (kg)/height squared (m^2^). Laboratory results obtained during routine clinical care were retrieved from the hospital information system. Nutritional indicators included serum albumin, prealbumin, hemoglobin (Hb), blood urea nitrogen (BUN), and total lymphocyte count (TLC). Serum albumin, prealbumin, Hb, and BUN levels had been analyzed using an automated biochemical analyzer (Mindray Bio-Medical Electronics Co., Ltd., Shenzhen, China; Model: BS-360E), while TLC had been determined using flow cytometry (Shenzhen Wellgrow Biotech Co., Ltd., Shenzhen, China; Model: EasyCell 103L0).

#### Eating Disorders

The Eating Attitudes Test-26 (EAT-26) [16] scores at baseline and week 12 were retrieved from electronic medical records and were used to reflect the severity of eating disorder symptoms. The test consisted of 26 items, each scored from 0 to 3 points. The scoring method was as follows: always (3 points), usually (2 points), often (1 point), sometimes / rarely / never (0 point). EAT-26 was divided into three subscales: Dieting (39 points), Bulimia and Food Preoccupation (18 points), and Oral Control (21 points). The total score was 78 points. The 26th item was scored in reverse. A score of ≥20 points indicated the risk of eating disorder [17].

#### Emotional Regulation Ability

The Trait Meta-Mood Scale (TMMS) [18] at 4, 8 and 12 weeks after treatment were extracted from electronic medical records. The TMMS consists of 30 items rated on a 5-point Likert scale ranging from 1 (“strongly disagree”) to 5 (“strongly agree”). Total scores range from 30 to 150, with higher scores indicating stronger emotion regulation ability. The internal consistency reliability was 0.79.

#### Anxiety and Depression

Data on anxiety and depressive symptoms at baseline and at weeks 4, 8, and 12 after treatment were extracted from electronic medical records. Symptom severity had been routinely recorded in clinical practice using the GAD-7 scale [19] and the CDI-2 [20]. The GAD-7 consists of 7 items; each scored from 0 to 3. Total scores of 5–9 indicate mild anxiety, 10–14 indicate moderate anxiety, and ≥15 indicate severe anxiety [19]. The CDI-2 includes 28 items, each rated on a 3-point scale (0–2) according to symptom severity, with a total score ranging from 0 to 56. Higher scores indicate more severe depressive symptoms. CDI-2 score ≥20 indicates severe depression.

#### Safety Outcomes

Safety outcomes during the 12-week treatment period were retrospectively collected from electronic medical records, including the occurrence of refeeding syndrome, serious adverse events requiring medical intervention, and readmission due to worsening eating disorder symptoms or nutritional deterioration.

### Statistical Methods

Statistical analyses were performed using SPSS version 26.0 (IBM Corp., Armonk, NY, USA). Continuous variables with a normal distribution were expressed as mean ± standard deviation (SD). Comparisons between two groups were conducted using independent-samples *t*-tests, while within-group comparisons before and after treatment were performed using paired *t*-tests or repeated-measures analysis of variance (ANOVA), as appropriate. Categorical variables were presented as frequencies and percentages and analyzed using the chi-square test. All statistical tests were two-tailed, and a *p* value < 0.05 was considered statistically significant.

To further assess the independent association between EFT and BMI improvement, multiple linear regression analysis was additionally performed. BMI at week 12 was included as the dependent variable, while treatment group, baseline BMI, age, sex, and olanzapine use were entered as independent variables.

To further evaluate the magnitude and clinical relevance of treatment effects, mean changes from baseline to week 12 were calculated for key outcome measures, including BMI, EAT-26, TMMS, GAD-7, and CDI-2 scores. Between-group differences in change scores were expressed as mean differences with 95% confidence intervals (CIs). Effect sizes were additionally estimated using Cohen’s d.

## Results

### Comparison of Clinical Data Between the Two Groups

Before treatment, there were no significant differences between the two groups in terms of gender, age, disease duration, BMI, disease severity, and medication use, indicating that baseline data of the two groups were comparable. As shown in Table 1.

**Table 1.** **Comparison of baseline data between the two groups**.

Clinical characteristics		Control group (n = 60)	EFT group (n = 60)	$\chi^{2}$/*t*	*p*
Gender				0.076	0.782
	Male	8 (13.3)	7 (11.7)		
	Female	52 (86.7)	53 (88.3)		
Age (year)		15.8 ± 1.1	15.6 ± 1.3	–0.910	0.365
Disease duration (months)		11.4 ± 2.5	11.7 ± 2.8	0.619	0.537
BMI (kg/ m^2^)		16.25 ± 1.32	16.44 ± 1.38	0.771	0.442
Family structure				0.326	0.568
	Two-parent family	23 (38.3)	20 (33.3)		
	Single-parent family	37 (61.7)	40 (66.7)		
Olanzapine use				0.164	0.685
	Yes	18 (30.0)	16 (26.7)		
	No	42 (70.0)	44 (73.3)		
GAD-7 score				0.300	0.584
	≥15	29 (48.3)	32 (53.3)		
	<15	31 (51.7)	28 (46.7)		
CDI-2 score				0.043	0.835
	≥20	45 (75.0)	44 (73.3)		
	<20	15 (25.0)	16 (26.7)		

EFT, emotion-focused therapy; BMI, body mass index; GAD-7, Generalized Anxiety Disorder-7; CDI-2, Children’s Depression Inventory-2.

### BMI and Nutritional Indicators

Before treatment, there were no significant differences in BMI and various nutritional indicators between the two groups (*p* > 0.05). After treatment, BMI, albumin, prealbumin, Hb, BUN, and TLC levels in both groups were significantly increased, and the EFT group showed significant higher BMI and nutritional indicator levels than the control group (*p* < 0.05). As shown in Table 2.

**Table 2.** **BMI and nutritional indicators before and after treatment in the two groups (mean ± SD)**.

Indicators	Time	Control group (n = 60)	EFT group (n = 60)	*t*	*p*
BMI (kg/m^2^)	Before treatment	16.25 ± 1.32	16.44 ± 1.38	0.771	0.442
	After treatment	17.10 ± 2.15^*^	18.21 ± 2.23^*⁣**^	2.776	0.006
Albumin (g/L)	Before treatment	35.09 ± 4.30	34.83 ± 4.25	–0.333	0.740
	After treatment	37.50 ± 5.40^**^	40.47 ± 6.10^*⁣**^	2.824	0.006
Prealbumin (mg/L)	Before treatment	175.50 ± 18.92	176.34 ± 20.13	0.236	0.814
	After treatment	188.04 ± 35.26^*^	205.18 ± 34.67^*⁣**^	2.685	0.008
Hb (g/L)	Before treatment	103.52 ± 11.15	105.70 ± 9.41	1.157	0.249
	After treatment	119.30 ± 9.43^*⁣**^	125.58 ± 9.48^*⁣**^	3.638	<0.001
BUN (mmol/L)	Before treatment	3.40 ± 0.98	3.41 ± 1.01	0.055	0.956
	After treatment	4.19 ± 1.27^*⁣**^	4.68 ± 1.31^*⁣**^	2.080	0.040
TLC (×10^9^/L)	Before treatment	1.32 ± 0.46	1.38 ± 0.43	0.738	0.462
	After treatment	1.96 ± 0.54^*⁣**^	2.50 ± 0.66^*⁣**^	4.905	<0.001

EFT, emotion-focused therapy; BMI, body mass index; Hb, hemoglobin; BUN, blood urea nitrogen; TLC, total lymphocyte count. Within-group comparison, ^*^p < 0.05, ^**^p < 0.01, and ^*⁣**^p < 0.001.

### Eating Disorders

Before treatment, there were no significant differences in the total EAT-26 score and scores of each subscale between the two groups (*p* > 0.05). After treatment, the total EAT-26 score and scores of each subscale in both groups were significantly lower than before treatment, and the EFT group showed significantly lower scores than the control group (*p* < 0.05). As shown in Table 3.

**Table 3.** **Comparison of eating disorder scores before and after treatment in the two groups (scores, mean ± SD)**.

EAT-26	Time	Control group (n = 60)	EFT group (n = 60)	*t*	*p*
Dieting	Before treatment	18.25 ± 2.32	18.44 ± 2.38	0.443	0.659
	After treatment	10.46 ± 1.21^*⁣**^	8.64 ± 0.99^*⁣**^	–9.017	<0.001
Bulimia and food preoccupation	Before treatment	9.38 ± 1.61	9.47 ± 1.65	0.302	0.763
	After treatment	5.84 ± 0.89^*⁣**^	4.62 ± 0.96^*⁣**^	–7.219	<0.001
Oral control	Before treatment	10.53 ± 1.18	10.80 ± 1.22	1.232	0.220
	After treatment	4.34 ± 0.42^*⁣**^	3.80 ± 0.32^*⁣**^	–7.922	<0.001
Total	Before treatment	38.16 ± 2.73	38.71 ± 3.06	1.039	0.301
	After treatment	20.64 ± 2.16^*⁣**^	17.06 ± 2.08^*⁣**^	–9.248	<0.001

EFT, emotion-focused therapy; EAT-26, Eating Attitudes Test-26. Within-group comparison, ^*⁣**^p < 0.001.

### Emotion Regulation and Anxiety/Depression Level

Before treatment, there were no significant differences in TMMS, GAD-7, and CDI-2 scores between the two groups (*p* > 0.05). At weeks 4, 8, and 12 after treatment, TMMS scores in both groups were higher than before treatment, with the EFT group showing higher scores than the control group. Conversely, GAD-7 and CDI-2 scores in both groups were lower than before treatment, with the EFT group showing lower scores than the control group. These differences were all statistically significant (*p* < 0.05). As shown in Table 4.

**Table 4.** **Emotion regulation and anxiety/depression scores before and after treatment in the two groups (scores, mean ± SD)**.

Emotion scale	Time	Control group (n = 60)	EFT group (n = 60)	*t*	*p*
TMMS	Before treatment	61.8 ± 9.2	62.3 ± 8.5	0.309	0.758
	Week 4	68.3 ± 10.3	74.2 ± 10.4	3.122	0.002
	Week 8	76.8 ± 9.7	86.1 ± 9.6	5.278	<0.001
	Week 12	88.5 ± 8.1	98.4 ± 7.6	6.904	<0.001
ANOVA F, *p*		91.163, <0.001	174.964, <0.001		
GAD-7	Before treatment	14.9 ± 3.1	15.0 ± 2.8	0.185	0.853
	Week 4	12.8 ± 2.8	11.6 ± 2.3	–2.565	0.012
	Week 8	10.7 ± 1.6	8.4 ± 1.3	–8.642	<0.001
	Week 12	8.5 ± 1.2	6.0 ± 1.1	–11.896	<0.001
ANOVA F, *p*		84.615, <0.001	228.921, <0.001		
CDI-2	Before treatment	25.4 ± 3.6	25.1 ± 3.7	–0.450	0.653
	Week 4	22.7 ± 3.4	20.3 ± 3.8	–3.646	<0.001
	Week 8	18.1 ± 2.3	15.9 ± 2.4	–5.126	<0.001
	Week 12	15.3 ± 2.5	10.7 ± 2.3	–10.489	<0.001
ANOVA F, *p*		136.633, <0.001	231.547, <0.001		

EFT, emotion-focused therapy; TMMS, Trait Meta-Mood Scale; ANOVA, analysis of variance; GAD-7, Generalized Anxiety Disorder-7; CDI-2, Children’s Depression Inventory-2.

### Safety Outcomes

No serious adverse events occurred in either group during the 12-week treatment period. Refeeding syndrome was observed in 3 patients (5.0%) in the control group and 2 patients (3.3%) in the EFT group, all of whom improved after electrolyte correction and nutritional adjustment, and showing no significant difference ($\chi^{2}$ = 0.000, *p* = 1.000). In addition, 6 patients (10.0%) in the control group and 2 patients (3.3%) in the EFT group required readmission, with no significant differences were observed between the two groups ($\chi^{2}$ = 1.205, *p* = 0.272).

### Multiple Linear Regression Analysis

Multiple linear regression analysis showed that EFT remained independently associated with higher BMI at week 12 after adjusting for baseline BMI, age, sex, and olanzapine use (β = 0.382, *p* < 0.001). (Table 5)

**Table 5.** **Multiple linear regression analysis of factors associated with BMI at week 12**.

Variable	B	SE	β	*t*	*p*
Constant	8.742	2.315	—	3.776	<0.001
Treatment (EFT = 1, control = 0)	1.286	0.241	0.382	5.336	<0.001
Baseline BMI	0.593	0.087	0.521	6.816	<0.001
Age	–0.071	0.064	–0.067	–1.109	0.270
Sex (female = 1, male = 0)	0.318	0.372	0.048	0.855	0.394
Olanzapine use (yes = 1, no = 0)	0.524	0.228	0.161	2.298	0.023

Model statistics: R^2^ = 0.487, adjusted R^2^ = 0.465, F = 22.731, *p* < 0.001. EFT, emotion-focused therapy; BMI, body mass index; SE, standard error.

### Effect Sizes for Major Outcomes

The EFT group demonstrated significantly greater improvements in BMI, eating behaviors, emotional regulation, anxiety, and depressive symptoms compared with the control group, with moderate-to-large effect sizes (Cohen’s d = 0.77–1.23). (Table 6)

**Table 6.** **Mean changes from baseline to week 12 and effect sizes between groups**.

Outcome	Control group Δ	EFT group Δ	Mean difference (95% CI)	Cohen’s d	*p*
BMI (kg/m^2^)	0.85 ± 1.12	1.77 ± 1.26	0.92 (0.49 to 1.35)	0.77	<0.001
EAT-26 total score	–17.52 ± 3.41	–21.65 ± 3.76	–4.13 (–5.42 to –2.84)	1.15	<0.001
TMMS	26.7 ± 10.1	36.1 ± 10.4	9.4 (5.7 to 13.1)	0.91	<0.001
GAD-7	–6.4 ± 2.5	–9.0 ± 2.6	–2.6 (–3.5 to –1.7)	1.02	<0.001
CDI-2	–10.1 ± 3.4	–14.4 ± 3.6	–4.3 (–5.6 to –3.0)	1.23	<0.001

Δ represents the change from baseline to week 12. EFT, emotion-focused therapy; BMI, body mass index; EAT-26, Eating Attitudes Test-26; TMMS, Trait Meta-Mood Scale; GAD-7, Generalized Anxiety Disorder-7; CDI-2, Children’s Depression Inventory-2; CI, confidence interval.

## Discussion

Adolescence is a high-risk period for AN, with a peak age of onset between 12 and 18 years. The disorder is more prevalent in females than in males. Pubertal changes in body shape, fluctuations in hormone levels, and sociocultural attention to weight and appearance are considered important contributing factors [21]. Compared with adult patients, adolescents exhibit greater emotional plasticity and tend to have a better prognosis when treated with psychological interventions [22]. However, there is currently no universally recognized optimal treatment.

EFT is an evidence-based, experiential therapeutic approach. It posits that emotions are shaped through the interaction between the self and the environment and aims to enhance the self as a means of improving emotional regulation. By addressing maladaptive emotional cycles characterized by loneliness, fear, and low self-esteem, EFT has important clinical value in the treatment of complex psychological disorders such as AN and bulimia nervosa in adolescents [23].

The results of this study show that EFT has a significant effect on weight restoration and improvement of nutritional indicators in adolescents with AN, and can also effectively reduce EAT-26 scores. This may be because EFT can improve the pathological emotional state of patients, promote changes in their nutritional intake behavior, effectively reduce negative behaviors such as self-induced vomiting or purging, promote nutritional intake, improve children’s understanding of dietary nutrition, promote a balanced diet, ensure daily calorie and protein intake, thereby improving nutritional indicator levels and promoting weight restoration [23]. Aarnio-Peterson *et al*. [24] reported that family-based emotional therapy can effectively promote weight recovery in adolescents with AN, consistent with the findings of this study.

In terms of emotion regulation ability, patients scored significantly higher on the TMMS scale after treatment, indicating that EFT effectively improved their emotion regulation ability by enhancing their awareness, acceptance and expression of their own emotional experiences. According to a review exploring EFT for AN, this improvement may be due to the application of “focusing techniques” and “empathy techniques” in EFT, which help patients overcome loneliness, gain a sense of efficacy and promote their emotional transformation [25]. Baudinet *et al*. [26] demonstrated that family-based emotional therapy can also promote the improvement of interpersonal and family relationships of children with AN, enabling them to receive more care and supervision, thereby shifting their focus away from appearance and alleviating anorexia symptoms. Another study on emotion regulation therapies has also shown that regulating the cognitive and emotional systems can alleviate the symptoms of mental illness, which provides further theoretical support for the mechanism of action of EFT [25].

Furthermore, effect size analysis demonstrated moderate-to-large treatment effects of EFT on BMI, eating behaviors, emotional regulation, anxiety, and depressive symptoms, suggesting that the observed improvements were not only statistically significant but also clinically meaningful. Multiple linear regression analysis further showed that EFT intervention remained independently associated with higher BMI at week 12 after adjustment for baseline BMI, age, sex, and olanzapine use. These findings indicate that the beneficial effects of EFT on weight restoration may not merely reflect baseline differences or concomitant pharmacological treatment but may be related to its role in improving emotional processing and reducing maladaptive eating-related cognitions and behaviors.

Regarding safety outcomes, no significant difference was observed in the incidence of refeeding syndrome between the two groups. The pathophysiological basis of refeeding syndrome involves the rapid transition from an adaptive catabolic state to an anabolic state after nutritional intake is restored in patients with prolonged malnutrition [27]. This process may lead not only to electrolyte disturbances, but also to impaired cardiac and neurological function [28]. Because refeeding syndrome is primarily associated with nutritional rehabilitation strategies, EFT, as a psychological intervention, was unlikely to substantially influence its incidence.

In addition, the readmission rate was lower in the EFT group (3.3%) than in the control group (10.0%), although the difference did not reach statistical significance, possibly due to the limited sample size. Readmission in adolescents with AN is commonly related to relapse and recurrence of restrictive eating behaviors. As a psychotherapy targeting emotional processing and regulation, EFT may help reduce negative emotional states, improve emotional regulation abilities, and alleviate psychological triggers associated with disease recurrence, thereby potentially reducing the risk of readmission.

While this study preliminarily validated the effectiveness of EFT in treating adolescents with AN, certain limitations remain. First, regarding sample selection, the sample size was small and from a single source, primarily focusing on adolescents in a specific region, which may affect the generalizability and extrapolation of the results. Second, the treatment lasted only for 12 weeks, while AN, as a chronic and easily relapsing mental illness, typically requires a longer period to observe long-term effects. Future research could extend the follow-up period to 6–12 months or increase the sample size to improve the representativeness and generalizability of the results.

Furthermore, retrospective observational trials are subject to limitations such as recall bias and selection bias, suggesting that future research should conduct prospective randomized controlled clinical trials to more accurately assess the lasting effects of EFT on patients’ weight restoration and emotion regulation. Additionally, although the TMMS was used to assess emotion regulation ability, only total TMMS scores were consistently available in the medical records, whereas subscale scores were not routinely documented. Future prospective studies with standardized assessments are warranted to further explore the mechanisms underlying the therapeutic effects of EFT in adolescents with AN.

## Conclusions

EFT combined with routine nutritional support and psychotherapy may be associated with improved weight restoration, emotion regulation, and nutritional status in adolescents with AN, as well as reduced anxiety and depression levels. However, due to the retrospective design, relatively small sample size, and single-center setting, causal relationships cannot be definitively established. Further large-scale and prospective trials are needed to validate the therapeutic effects of EFT in adolescents with AN.

## Availability of Data and Materials

The datasets used and/or analysed during the current study were available from the corresponding author on reasonable request.
